# Pelvic girdle pain in pregnancy and early postpartum – prevalence and risk factors in a multi-ethnic cohort

**DOI:** 10.1186/s12891-023-07135-w

**Published:** 2024-01-02

**Authors:** Hilde Stendal Robinson, Nina K. Vøllestad, Karin Elisabeth Bennetter, Christin W. Waage, Anne Karen Jenum, Kåre Rønn Richardsen

**Affiliations:** 1 Department of Interdisciplinary Health Sciences, Institute of Health and Society, University of Oslo, Blindern, P.O. Box 1089, 0317 Oslo, Norway; 2 Department of General Practice, Institute of Health and Society, University of Oslo, Oslo, Norway; 3 Department of Rehabilitation Science and Health Technology, Faculty of Health Science, Oslo Metropolitan University, Oslo, Norway; 4 General Practice Research Unit (AFE), Institute of Health and Society, Department of General Practice, University of Oslo, Oslo, Norway; 5 Department of Rehabilitation Science and Health Technology, Faculty of Health Science, Oslo Metropolitan University, Oslo, Norway

**Keywords:** Ethnicity, Pregnancy, Population-based study, Longitudinal study, Questionnaire-data

## Abstract

**Background:**

Pelvic girdle pain (PGP) is common during and after pregnancy. It has been assumed that Scandinavian women report more PGP than women of other ethnicities. However, there are few population-based studies on ethnic differences and few with ethnicity as risk factor for PGP. The purposes of the present study were: To examine the prevalence of self-reported PGP through pregnancy and early postpartum in a multi-ethnic cohort. To investigate how ethnicity and patient characteristics were associated with risk of PGP during pregnancy and early postpartum. To investigate if clinical and personal factors obtained in gestation week (GW) 15 were associated with PGP in GW28 and postpartum week (PPW) 14.

**Methods:**

This study analyzed questionnaire data from 823 women from the Stork - Groruddalen mult-iethnic cohort study in Norway. Chi-square tests were used to investigate ethnic differences in prevalence of self-reported PGP, and logistic regression analyses to identify factors associated with self-reported PGP.

**Results:**

Women from South-Asia and Middle East reported 10-20% higher prevalence of self-reported PGP at all time points compared with Western women. Ethnicity was associated with PGP in GW15 and PPW14, adjusted for parity. Pain locations in pelvic area (PGP locations) in GW15, especially combined symphysis and posterior PGP, gave the highest risk (OR=7.4) for PGP in GW28 and in PPW14 (OR = 3.9). Being multiparous was a risk for PGP in PPW14 (OR=1.9).

**Conclusions:**

Women of South Asian and Middle Eastern background had higher risk of self-reported PGP than Western women. Ethnicity was associated with PGP in GW15 and PPW14, after adjustments for parity. PGP locations in GW15 was the most prominent risk factor for PGP in GW28 and PPW14, whilst ethnicity was not significant in multivariable analyses.

**Supplementary Information:**

The online version contains supplementary material available at 10.1186/s12891-023-07135-w.

## Background

Pelvic girdle pain (PGP) is a common musculoskeletal complaint during and after pregnancy with reported prevalence between 7-65% during pregnancy and between 0-41% postpartum in studies from the past two decades [1–8]. Possible explanations for this large variation may be inconsistent terminology, cultural differences, data collection in different gestation week (GW) and different number of postpartum weeks (PPW) as well as other methodological differences. Some studies used self-reports of pain based on questions and/or pain drawings [3, 4, 9], while others used clinical examination to classify PGP [1, 10]. Different definitions of PGP may also have introduced variation in PGP prevalence across studies [5, 11, 12]. However, it is commonly accepted that women with combined pain in the symphysis pubis and posterior parts of the pelvis (sacroiliac joints) are more afflicted [2, 10, 13] and have poorer prognosis [13]. In addition to possible methodological issues, the selected study samples or populations studied may also contribute to variation in results.

It has been a commonly accepted assumption that Scandinavian pregnant women report PGP more often than women in other countries [5]. Moreover, there are studies from a wide number of countries across continents, indicating that PGP is a worldwide problem [5]. Nevertheless, there are few population-based studies of ethnic differences in prevalence of PGP, and few have included ethnicity as a risk factor for PGP in their analyses. Ceprnja and co-workers reported that country of birth was a risk factor for PGP in an Australian study of pregnant women [4]. Starzec and co-workers compared the prevalence of PGP in two samples of pregnant women, one living in Poland and one in Norway using pain drawings to identify pain location [9]. They found no statistical difference in the prevalence of PGP, but a larger proportion of the Norwegian women reported combined symphysis and posterior PGP as well as combined PGP and low back pain (lumbopelvic pain) [9].

We have previously studied PGP in two cohorts in Norway [2, 10], and found prevalence between 35 and 62%, depending on the methods used and time in pregnancy. Since being fluent in the Norwegian language was one of the inclusion criteria in both studies, only small numbers of participants of other ethnicity than Norwegian women participated. Hence, these studies could give no valid information about PGP in immigrant or ethnic minority women in Norway. In European countries it is an issue that immigrants are underrepresented in studies since most studies have language proficiency as an inclusion criterion [14]. Not speaking the country’s majority language, low health literacy or general mistrust often reduce their participation in health surveys [14, 15]. Moreover, adaptation of recruitment strategies and methodology to reach these groups may increase the research costs considerably. The population-based STORK-Groruddalen cohort study included pregnant women of different ethnicity living in Oslo, Norway, independent of their proficiency in the Norwegian language [16]. The study was primarily set up to examine gestational diabetes, physical activity, and obesity during pregnancy in a multi-ethnic population. However, questions about PGP and location of the pain in the pelvic area, as well as information about potential risk factors for PGP were obtained from questionnaires at three timepoints (GW15, GW28 and 14 weeks postpartum (PPW14)).

The present study had the following three aims: 1) to examine the prevalence of PGP through pregnancy and early postpartum in a multi-ethnic cohort in Norway, 2) to investigate how ethnicity and patient characteristics were associated with risk of PGP during pregnancy and early postpartum, 3) to investigate if clinical and personal factors obtained in GW15 were associated with PGP in GW28 and PPW14.

## Methods

The present study used data from the population based STORK-Groruddalen study [16, 17], performed in accordance with the Declaration of Helsinki, and approved by the Regional Committee for Medical and Health Research Ethics in Norway (ref: 2007/894) and the Norwegian Data Inspectorate. All participants signed written informed consent before inclusion. In Norway, the public health services offer all women free health services and routinely scheduled controls at specific timepoints (GWs) of mother and fetus through pregnancies. Women attend their general practitioner and/or a midwife at the Child Health Care clinics (CHCs) for follow-up in their pregnancies. A total of 823 pregnant women attending the CHCs in Groruddalen in Oslo (including Stovner, Grorud and Bjerke city districts) for antenatal care, participated in the present study. The data-collection period was 2008 -2011 and the total population in these districts was about 82 500 people with diverse ethnic background and socioeconomic position.

### Data collection procedure

Trained midwives collected data at the CHCs, using interviewer-administrated questionnaires according to protocol and were available to solve eventual unclarities with the questions, pain locations or other issues [16]. To facilitate participation of ethnic minority women, all information material and questionnaires were translated to eight languages: Arabic, English, Sorani, Somali, Tamil, Turkish, Urdu, and Vietnamese, covering the largest ethnic groups. The translations were performed by the City Services Department, The Interpreting and Translating Service in Oslo by two professional translators for each language and thereafter quality checked by bilingual health professionals. Professional interpreters were available at the CHCs, if needed, and were used by about 22% of the participants [16].

### Outcome variables

In this study, the presence of PGP was based on questions about having some or much pain in the symphysis pubis alone, pain in one or both posterior pelvic joints, and combined pain in the symphysis and the posterior pelvic joints (with three response alternatives: no – yes/some - yes/a lot of pain). Response on these questions (yes) were categorized into *PGP locations* as follows: no pain, symphysis pain only, posterior PGP only, combined symphysis and posterior PGP [1, 3, 18]. We calculated prevalence of PGP pain by location at three time points (at inclusion (GW15), in GW28 and PPW14) for all women.

Furthermore, reporting some or much pain in at least one of the pain locations were coded as “yes”, and reporting no pain as “no”. This binary measure of PGP (yes/no) was used as outcome variable for the logistic regression analyses.

### Explanatory variables

The main explanatory variable was ethnicity. Based on the information about the participants’ country of birth, or the country of birth of the participant’s mother if the mother was born outside Europe or North America, groups of ethnicities were defined and used as in previous publications from the Stork Groruddalen study [16, 19, 20]. Hence, ethnicity was categorized as Western, South Asian, Middle Eastern, and Mixed ethnic group. The Mixed ethnic group was highly heterogeneous with few women from each country (Supplementary Table S1). Most women in the Western group were ethnic Norwegian (313 out of 336, 93%). Western women were used as reference in the regression models.

The following variables were explored as possible risk factors for PGP in the logistic regression analyses [4, 11, 21–24] : age, parity (nulli-, primi-, multiparous) self-rated health (very good, good, poor – not so good), depressive symptoms (measured by Edinburgh postnatal depression scale (EDPS)>10 (yes, no), and change in self-reported level of physical activity from pre-pregnancy (unchanged, less active, more active). We also investigated if PGP locations (described above) in GW15 was associated with having PGP in GW 28 and in PPW14 [18].

### Statistical analyses

Descriptive data are presented as frequencies with percentages (%) and mean with standard deviation (SD). To compare the prevalence of PGP in the different ethnic groups, we used Chi-square tests. We also calculated the number of women reporting to have much pain at each of the three timepoints.

To study the association between ethnicity and PGP (any location, yes/no) in pregnancy (in GW15 and GW28) and PPW14, we built logistic regression models and presented the associations as crude and adjusted odds ratios (OR) with 95% Confidence Intervals (CIs). Parity, education, and age were included in these multivariable analyses based on previous studies [25].

Factors associated with risk of PGP in GW28 and PPW14 were investigated using logistic regression analyses and presented as crude and adjusted ORs with 95% CIs. All variables significantly associated with PGP in the univariate regression analyses were entered in the multivariable logistic regression models. Non-significant variables with the highest *p*-values were excluded one by one until the model had only significant variables. Multicollinearity was examined using Variance Inflation Factor (VIF).

Statistical significance level for the analyses was set at *p*<0.05, with exception for the univariate logistic regression analyses, where *p* ≤ 0.1 were used. All analyses were performed in SPSS version 27.

## Results

Of the 823 pregnant women participating in this study, 46%, 34% and 20% were nulliparous, primiparous and multiparous, respectively. Mean age (SD) was 30 (5) years (Table 1). Moreover, 41% of the participants were Western, whilst 24% and 15% were categorized as South Asian and Middle Eastern, respectively. The remaining 20% were in the Mixed ethnic group. The four ethnic groups were comparable for most descriptive data, but a lower proportion of Western women were multipara and a larger proportion had higher education compared with the other groups (Table 1).

**Table 1:** Characteristics of the cohort (*n*=823) of pregnant women by ethnic groups. Values in mean and standard deviation (SD), numbers and percent (%)

		Total *n*=823 (100%)	Western *n*=336 (41)	South Asia *n*=200 (24)	Middle East *n*=126 (15)	Mixed ethnic group *n*=161 (20)
Mean age (SD) at inclusion, gestation week 15		30 (5.0)	31 (4.5)	29 (4.5)	29 (5.5)	29 (5.0)
Mean BMI (SD), from self-reported weight at inclusion		25.3 (4.9)	25.3 (4.7)	24.4 (4.2)	26.9 (5.4)	25.4 (5.3)
Parity, n (%)	Nulliparous	381 (46)	176 (52)	84 (42)	44 (35)	77 (48)
	Primiparous	280 (34)	125 (37)	65 (32)	43 (34)	47 (29)
	Multiparous	162 (20)	35 (11)	51(26)	39 (31)	37 (23)
Education, years, n (%)	Primary or less	133 (16)	10 (3)	35 (17)	46 (37)	42 (26)
	High school/secondary	324 (39)	103 (31)	101 (51)	55 (44)	65 (41)
	College/University	360 (44)	221 (66)	63 (32)	23 (18)	53 (33)
Married/cohabitant, yes (%)		776 (94)	320 (95)	197 (99)	121 (96)	138 (86)
EPDS-score >10 (gestation week 28), yes (%)		96 (12)	25 (7)	33 (17)	22 (17)	16 (10)
EPDS-score>10 14 weeks postpartum, yes (%)		60 (7)	12 (4)	18 (9)	17 (14)	11 (7)

Mixed ethnic group: mainly from Eastern Europe, East Asia, South and Central America, and Africa (Supplementary Table 1)

*BMI* Body Mass Index, *EPDS* Edinburgh Postnatal Depression Score

### Prevalence of PGP during pregnancy and PPW14

In GW15 and GW28, a total of 42% and 62% of all participants reported pain in one or more of the pelvic joints respectively. In PPW14 the prevalence was 27% (Table 2). Most of the women reported to have some pain, but 13%, 24% and 18% of the women with PGP reported to have much pain in GW15, GW28 and PPW14, respectively.

**Table 2:** Pelvic girdle pain (PGP) locations by ethnic groups in gestation week (GW) 15, 28 and 14 weeks postpartum

	PGP (yes) any location n (%)	No pain n (%)	Pain in Symphysis Pubis only n (%)	Posterior PGP only n (%)	Combined symphysis and posterior PGP n (%)	Total n [numbers missing]
***Gestation week 15***	***335 (42)***	***463 (58)***	***65 (8)***	***120 (15)***	***150 (19)***	***798 [25]***
Western	103 (32)	222 (68)	16 (5)	52 (16)	35 (11)	325
South Asia	100 (51)	96 (49)	13 (7)	34 (17)	53 (27)	196
Middle East	69 (51)	55 (44)	19 (15)	17 (14)	33 (27)	124
Mixed ethnic group	63 (41)	90 (59)	17 (11)	17 (11)	29 (19)	153
***Gestation week 28***	***463(62)***	***286 (38)***	***87 (12)***	***145 (19)***	***231 (31)***	***749 [74]***
Western	169 (56)	134 (44)	31 (10)	59 (19)	79 (26)	303
South Asia	130 (68)	61 (32)	26 (8)	33 (17)	71 (37)	191
Middle East	79 (70)	34 (30)	23 (20)	23 (20)	33 (29)	113
Mixed ethnic group	85 (60)	57 (40)	7 (5)	30 (21)	48 (34)	142
***14 weeks postpartum***	***177 (27)***	***473 (73)***	***26 (4)***	***60 (9)***	***91 (14)***	***650 [173]***
Western	60 (22)	217 (78)	12 (4)	21 (8)	27 (10)	277
South Asia	60 (38)	98 (62)	9 (6)	17 (11)	34 (22)	158
Middle East	29 (30)	67 (70)	4 (4)	10 (10)	15 (16)	96
Mixed ethnic group	28 (24)	91 (76)	1 (.8)	12 (10)	15 (13)	119

Mixed ethnic group: women mainly from Eastern Europe, East Asia, South and Central America, and Africa (See Table S1 in Supplementary)

The South-Asian and Middle Eastern women reported the highest prevalence of PGP (any location) at all three timepoints, 10-20% higher than the Western women (Table 2).

### Associations between ethnicity, patient characteristics, and PGP in GW15, GW28 and PPW14

In the univariate logistic analyses, ethnicity was associated with PGP (yes/no) at all three time points. In the multivariable logistic regression analyses including patient characteristics, ethnicity and parity were associated with PGP at GW15 and parity was associated with PGP in PPW14 (Table 3, and model 1 in Table 5). Moreover, women from South Asia had significantly higher risk for PGP in GW15 and PPW14 (Table 3 and model 1 in Table 5) and women from the Middle East had significantly higher risk in GW15 compared with Western women (Table 3).

**Table 3:** Association between pelvic girdle pain (PGP) and personal factors (ethnicity, parity, education, and age) at gestation week 15 presented with crude and adjusted odds ratios (OR) with 95% confidence interval (CI)

		**Gestation week 15**			
		Crude OR (95% CI)	***p* -value**	**Adjusted OR (95% CI)**	***p*-value**
**Ethnicity**	Western	Reference	<0.001	Reference	<0.001
	South Asia	2.2 (1.5, 3.2)		2.0 (1.3, 2.9)	
	Middle East	2.7 (1.8, 4.2)		2.4 (1.5, 3.9)	
	Mixed ethnic group	1.5 (1.0, 2.2)		1.3 (0.9, 2.1)	
**Parity**	Nulliparous	Reference	<0.001	Reference	0.006
	Primiparous	1.3 (0.9, 1.8)		1.4 (0.97, 1.9)	
	Multiparous	1.7 (1.1, 2.6)		2.0 (1.3, 3.2)	
**Education**	Primary or less	Reference	0.042	Reference	0.694
	High school/secondary	0.9 (0.6, 1.4)		1.2 (0.8, 1.9)	
	College/University	0.65 (0.43, 0.98)		1.2 (0.7, 1.9)	
**Age**		0.99 (0.96, 1.02)	0.531	1.0 (0.9, 1.0)	0.248

Mixed ethnic group: women mainly from Eastern Europe, East Asia, South and Central America, and Africa (Supplementary Table 1)

### Risk factors for reporting PGP in GW28

When we examined risk factors for PGP (yes/no) in GW28 we found that ethnicity, parity, PGP locations in GW15, self-rated health in GW15 and physical activity level were associated with PGP in the univariate analyses. Age, pre-pregnancy BMI, depressive symptoms (EDPS>10) and education were not associated, and not included in the multivariable analyses. In the multivariable logistic regression analyses, PGP locations in GW15, and especially combined symphysis and posterior PGP, gave the highest OR (7.4) for PGP in GW28 (model 2, Table 4). Being less active in GW15 than before pregnancy was a significant risk factor for PGP in GW28, with OR 1.6 (model 2, Table 4). No multicollinearity was found, all VIFs ≤1.23.

**Table 4:** Associations between Pelvic Girdle Pain (PGP) (yes/no) and risk factors at gestation week 28, presented with crude and adjusted odds ratios (OR) with 95% confidence interval (CI). Two final models are presented: in Model 1 the focus is on the association between Ethnicity, PGP and parity. In Model 2 the focus is on risk factors for PGP

	**Gestation week 28**			**Model 1**		**Model 2**	
	Total *n*=706	Crude OR (95% CI)	*p* -value	Adjusted OR (95% CI)	*p*-value	Adjusted OR (95% CI)	*p*-value
**Ethnicity**	Western (290)	Reference	0.01	Reference	0.123	-	
	South Asia (178)	1.7 (1.2, 2.5)		1.5 (0.98, 2.2)			
	Middle East (109)	1.8 (1.2, 1.9)		1.7 (0.98, 2.7)			
	Mixed ethnic group (129)	1.2 (0.8, 1.8)		1.1 (0.72, 1.7)			
**Parity**	Nulliparous (326)	Reference	0.03	Reference	0.014	-	-
	Primiparous (241)	1.3 (0.9, 1.9)		1.4 (1.01, 2.0)			
	Multiparous (139)	1.7 (1.1, 2.6)		1.9 (1.2, 3.1)			
**PGP locations in GW15**	No pain (410)	Reference	<0.001			Reference	<0.001
	Symphysis pain (59)	2.6 (1.5, 4.8)				3.0 (1.6, 5.5)	
	Posterior PGP (107)	2.7 (1.7, 4.6)				2.8 (1.8, 4.5)	
	Combined symphysis and posterior PGP (130)	6.9 (4.1, 11.8)				7.4 (4.2, 13.0)	
**Self-rated health in GW15**	Very good (274)	Reference	0.002			-	-
	Good (357)	2.6 (1.5, 4.7)					
	Poor, not really good (75)	1.4 (1.03, 1.9)					
**Physical activity level GW15**	Unchanged from pre-pregnancy (174)	Reference	0.02			Reference	0.013
	Less active (506)	1.5 (1.1,2.2)				1.6 (1.1, 2.3)	
	More active (26)	0.8 (0.4, 1.7)				0.7 (0.3, 1.7)	

Mixed ethnic group: women mainly from Eastern Europe, East Asia, South and Central America, and Africa (See Table S1 in Supplementary)

Education and age were not associated in the univariate analyses, and not entered in any of the two multivariable models. Ethnicity was no longer associated in the multivariable model 2

*GW* gestation week

### Risk factors for reporting PGP in PPW14

In the univariate logistic regression analyses ethnicity, parity, PGP locations in GW15, depressive symptoms in GW28 (EDPS>10) were associated with PGP in PPW14. Age, BMI, and physical activity level were not associated with PGP, and not included in multivariable models. In the multivariable logistic regression analyses, PGP locations in GW15, especially having combined symphysis and posterior PGP (OR = 3.9) and being multiparous (OR 1.9) were associated with having PGP in PPW14 (model 2 Table 5). No multicollinearity was found, all VIFs ≤1.04.

**Table 5:** Associations between Pelvic Girdle Pain (PGP) (no/yes) and risk factors at postpartum week 14 presented with crude and adjusted odds ratios (OR). Two final models are presented: in Model 1 the focus is on Ethnicity and personal factors and in Model 2 the focus is at risk factors for PGP

	**Postpartum week 14**			**Model 1**		**Model 2**	
		Crude OR (95% CI)	*p*-value	Adjusted OR (95% CI)	*p*-value	Adjusted OR (95% CI)	*p*-value
**Ethnicity**	Western	Reference	0.002	Reference	0.006	-	
	South Asia	2.2 (1.4, 3.4)		2.3 (1.4, 3.7)			
	Middle East	1.6 (0.9, 2.6)		1.5 (0.8, 2.7)			
	Mixed ethnic group	1.1 (0.7, 1.9)		1.1 (0.6, 1.9)			
**Parity**	Nulliparous	Reference	<0.001	Reference	0.001	Reference	<0.001
	Primiparous	0.9 (0.6, 1.3)		0.8 (0.5, 1.3)		0.7 (0.5, 1.1)	
	Multiparous	2.3 (1.5, 3.5)		2.0 (1.15, 3.3)		1.9 (1.2, 3.0)	
**PGP locations at GW15**	No pain (352)	Reference	<0.001			Reference	<0.001
	Symphysis (50)	2.2 (1.2, 4.1)				2.0 (1-1, 3.8)	
	Posterior PGP (94)	2.0 (1.2, 3.2)				2.0 (1.1, 3.1)	
	Combined symphysis and posterior PGP (109)	4.1 (2.6, 6.4)				3.9 (2.5, 6.2)	
**EDPS>10 at GW28**	Yes (79)	1.9 (1.2, 3.1)	0.008			-	-

Mixed ethnic group: women mainly from Eastern Europe, East Asia, South and Central America, and Africa (See Table S1 in Supplementary)

Age, Body Mass Index (BMI) as well as physical activity level were not significantly associated with PGP postpartum in the univariate analyses and was neither included in the multivariable analyses in Model 1 nor in Model 2. Ethnicity and scores on Edinburgh Postnatal Depression scale at GW28 was associated with PGP 14 weeks postpartum in the univariate analyses but not in the multivariable model 2

*GW* gestation week, *EDPS* Edinburgh Postnatal Depression scale

## Discussion

In this multiethnic cohort study of PGP during and after pregnancy, we found ethnic differences in prevalence of PGP at each timepoint. We found higher risk of PGP among women from South Asia and the Middle East compared with Western women. When we explored risk factors for PGP in GW28 and in PPW14 and included PGP locations as well as clinical factors in the analyses, ethnicity was no longer a significant risk factor, and having combined symphysis and posterior PGP in GW15 was the characteristic most strongly associated with PGP at both time points.

### Prevalence of PGP among different ethnic groups

Surprisingly, we found higher prevalence of PGP among South Asian women, women from the Middle East and women in the Mixed ethnic group compared to the Western women, at GW15 and GW28. Moreover, the Non-Western groups reported higher prevalence of PGP compared to previous published Norwegian data [2, 18], with predominantly Western participants. Somewhat different results were found postpartum, where a high prevalence of PGP was observed in the South Asian women (38%) in particular. The prevalence of combined symphysis and posterior PGP was between 26% (Western women) and 37% (South Asian women) in GW28. This is considerably higher than the 15% combined symphysis and posterior PGP in late pregnancy found in the Norwegian mother and child (MoBA) study [11]. This discrepancy can probably be explained by methodological differences and the registration of pain location (i.e., different questions). In our study, large efforts were made to include immigrant women. Due to this, the number of women in the ethnic groups are probably overrepresented, since the distribution of pregnant immigrant women differs between municipalities in Norway [26]. Yet, we can assume that the prevalence of PGP within the defined groups (Western, South Asian and Middle eastern) is representative for the women living in urban parts of Norway [16, 27]. The data used in this study was collected some years ago, but the prevalence and associations are in accordance with results from subsequent studies and a systematic review [4, 7, 28].

### Associations between PGP, ethnicity, and other patient characteristics in GW15, GW28 and PPW14

The ethnic differences in PGP were largest in the univariate logistic analyses and were reduced when we adjusted for parity in the multivariable logistic regression analyses (Table 3, model 1 in Tables 4 and 5). Moreover, the different ethnic groups were comparable in most descriptive factors (Table 1) except that a lower proportion of Western women were multiparous, and a larger proportion had higher education.

### Risk factors for PGP

When analyzing risk factors for PGP in GW28 and PPW14, we found that ethnicity was not significantly associated with PGP, when PGP locations in GW15 were included in the multivariable analyses. Reporting pain in any of the locations in GW15 was a risk factor for PGP in both GW28 and PPW14, and the largest risk was found for combined symphysis and posterior PGP. These observations are in accordance with results from previous studies [18, 29]. Moreover, having combined symphysis and posterior PGP has previously been found as an indication of more severe PGP [10, 13] and also associated with poorer prognosis [13]. Altogether this indicates that clinicians should be aware of women with any type of PGP in GW15, and especially women with combined symphysis and posterior PGP, since they have an increased risk of developing long-lasting PGP.

Age was not a risk factor for PGP in our study, and this is in accordance with the conclusions of the mini-review from Kanakaris and co-workers [5].

In contrast to a previous study [11], we did not identify parity as a risk factor for PGP in GW28. Since we studied risk factors for any location of PGP and not only for combined symphysis and posterior PGP (called pelvic girdle syndrome, PGS), the results cannot be compared directly. The number of participants were much larger in Bjellands study indicating higher power [11], and this probably explains why they found more significant factors. The study from Albert and co-workers [21] might be more comparable with ours, since they also reported risk for having PGP (any location). Moreover, parity, low back pain and trauma were risk factors for any PGP in pregnancy in their study. We found higher OR for PGP locations, than Albert did for LBP, indicating a stronger relationship, and might somewhat explain why parity was not a significant in our analysis. Previous published cohort studies also have results in accordance with ours in that having pain in any of the locations [18] and more disability [30] early in pregnancy were risk factors for PGP in late pregnancy and early postpartum.

We found that depressive symptoms (EDPS>10 in GW28) was a risk factor for PGP PPW14 only in the univariate and not in the multivariable logistic regression analyses. One previous study of risk factors for having combined symphysis and posterior PGP 6 months postpartum [24] found that depressive symptoms was a risk factor, but methodological differences hamper further comparison.

### Strengths and limitations

The strength of this population-based study is the ethnic diversity and success of including large numbers of immigrant women. This study shows that when more effort is directed towards strategies facilitating the inclusion of immigrants, such as questionnaires available in eight languages, use of trained interviewers and use of interpreters, it was possible to reduce barriers for participation, even for illiterate women, and to collect high quality data with few missing.

Due to resource limitations, the methodological quality of all questionnaires was not formally tested. The meaning of specific words such as pain might also differ across ethnic groups [15]. This might contribute to different interpretations of the questions. However, this is probably reduced by using interviewer-administered questionnaires, as well as midwives and interpreters available to solve unclarities. We cannot fully exclude that the use of interpreters might have been challenging and that mistrust can be present for some participants.

The data on PGP and pain location in the pelvic region are self-reported, and it can be criticized that no clinical examinations verified the self-reports. However, one previous study examined the associations between self-reported PGP locations, disability, and response on clinical tests and found that self-reported pain locations were significantly associated with clinical tests and influenced disability in pregnant women [10]. The women in the present study were recruited from the CHC’s, where they came for routine antenatal care. Hence, we neither have information about the number of the women seeking treatment for PGP, nor about degree of disability among the participants. However, the large proportion of women reporting to have some (and not much) pain could indicate that most of them are not treatment seeking. This information can be of importance when health authorities interpret prevalence numbers. Previous comparable population-based studies will have the similar uncertainty concerning verification and treatment, since none of them have included clinical examinations.

## Conclusion

In contrast to previous assumptions of higher prevalence of PGP among Western women compared with women of other ethnicity, our data show that women of South Asian and Middle Eastern background reported more PGP and had a higher risk of self-reported PGP than Western women. We found that ethnicity was associated with PGP in GW15 and PPW14, even after controlled for parity. Reporting pain in any location of the pelvis and especially combined symphysis and posterior PGP in GW15 was the most prominent risk factor for having PGP both in GW28 and PPW14, whilst ethnicity was no longer a significant risk factor when these factors were included in the models.

### Supplementary Information

**Additional file 1: Table S1.** Overview over classification of countries into group used in the analyses.

## Data Availability

The editors can access data in de-identified form used in the manuscript, code book, and analytical code upon request. Co-author Kåre Rønn-Richardsen will, together with the project manager Anja Maria Lyche Brænd (a.m.l.brand@medisin.uio.no), contribute to the access being provided under appropriate conditions. However, research data for this publication include identifying health information subject to confidentiality. It is therefore not possible to share raw data.

## Abbreviations

PGP: Pelvic girdle pain
GW: Gestation week
PPW: Postpartum week
CHC: Child health care clinic
EDPS: Edinburgh postnatal depression scale
SD: Standard deviation
OR: Odds ratio
CI: Confidence Interval
VIF: Variance Inflation Factor
BMI: Body mass index
MoBa: the Norwegian mother and child study
