# Supplementary material for: Pelvic girdle pain in pregnancy and early postpartum – prevalence and risk factors in a multi-ethnic cohort
Source: BMC Musculoskelet Disord. 2024 Jan 2;25:21. doi: 10.1186/s12891-023-07135-w (PMC10759664; doi:10.1186/s12891-023-07135-w)
Supplement: Supplementary file 1 — Additional file 1: Table S1. Overview over classification of countries into group used in the analyses. [file 12891_2023_7135_MOESM1_ESM.docx]

**Table S1.** Overview over classification of countries into group used in the analyses.

| Western | South Asia | Middle East | Mixed ethnic group | |
| --- | --- | --- | --- | --- |
| Canada  Denmark  France  Germany  Norway  Spain  Sweden  United Kingdom  USA | Bangladesh  India  Pakistan  Sri Lanka | Afghanistan  Algeria  Azerbaijan  Georgia  Iraq  Iran  Lebanon  Morocco  Palestine  Syria  Tunis  Turkey | Bosnia  Bulgaria  Burma  Chile  Columbia  Dominican Republic  Eritrea  Ethiopia  Philippines  Gambia  Ghana  Grenada  Cambodia  Cameroon  Kapp Verde  Kenya  China  Kongo  Korea  Kosovo  Croatia  Latvia | Lithuania  Macedonia  Mexico  Nigeria  Peru  Poland  Romania  Russia  Serbia  Singapore  Slovakia  Somalia  Surinam  Taiwan  Thailand  Togo  Czech Republic  Ukraine  Vietnam |

Mixed ethnic group contains miscellaneous group of countries. Several of the countries have very few participants.
